# The Role of Google Scholar in Evidence Reviews and Its Applicability to Grey Literature Searching

**DOI:** 10.1371/journal.pone.0138237

**Published:** 2015-09-17

**Authors:** Neal Robert Haddaway, Alexandra Mary Collins, Deborah Coughlin, Stuart Kirk

**Affiliations:** 1 MISTRA EviEM, Royal Swedish Academy of Sciences, Stockholm, Sweden; 2 Centre for Environmental Policy, Imperial College, London, United Kingdom; 3 Department for Environmental, Food and Rural Affairs, London, United Kingdom; 4 Department for Civil and Environmental Engineering, Imperial College, London, United Kingdom; 5 Environment Agency, London, United Kingdom; State University of New York, Oswego, UNITED STATES

## Abstract

Google Scholar (GS), a commonly used web-based academic search engine, catalogues between 2 and 100 million records of both academic and grey literature (articles not formally published by commercial academic publishers). Google Scholar collates results from across the internet and is free to use. As a result it has received considerable attention as a method for searching for literature, particularly in searches for grey literature, as required by systematic reviews. The reliance on GS as a standalone resource has been greatly debated, however, and its efficacy in grey literature searching has not yet been investigated. Using systematic review case studies from environmental science, we investigated the utility of GS in systematic reviews and in searches for grey literature. Our findings show that GS results contain moderate amounts of grey literature, with the majority found on average at page 80. We also found that, when searched for specifically, the majority of literature identified using Web of Science was also found using GS. However, our findings showed moderate/poor overlap in results when similar search strings were used in Web of Science and GS (10–67%), and that GS missed some important literature in five of six case studies. Furthermore, a general GS search failed to find any grey literature from a case study that involved manual searching of organisations’ websites. If used in systematic reviews for grey literature, we recommend that searches of article titles focus on the first 200 to 300 results. We conclude that whilst Google Scholar can find much grey literature and specific, known studies, it should not be used alone for systematic review searches. Rather, it forms a powerful addition to other traditional search methods. In addition, we advocate the use of tools to transparently document and catalogue GS search results to maintain high levels of transparency and the ability to be updated, critical to systematic reviews.

## Introduction

Searching for information is an integral part of research. Over 11,500 journals are catalogued by Journal Citation Reports (http://thomsonreuters.com/journal-citation-reports/), and the volume of published scientific research is growing at an ever-increasing rate [1,2]. Scientists must sift through this information to find relevant research, and do so today most commonly by using online citation databases (e.g. Web of Science) and search engines (e.g. Google Scholar). Just as the number of academic articles and journals is steadily increasing, so too are the number of citation databases.

A citation database is a set of citations that can be searched using an online tool, for example Web of Science (https://webofknowledge.com/). These databases typically charge subscription fees for access to the database that do not cover the cost of access to the full text of the research articles themselves. Generally these databases selectively catalogue citations according to a predefined list of journals, publishers or subject areas. Several free-to-use services have recently appeared that search for citations on the internet, most notably Google Scholar and Microsoft Academic Search. These search engines do not store citations within a specific database, instead they regularly ‘crawl’ the internet for information that appears to be a citation. Some key characteristics of databases and search engines are compared in Table 1.

**Table 1:** Typical characteristics of academic citation databases and search engines.

Feature	Academic Citation Databases	Academic Citation Search Engines
Time coverage	Depends on the database, but time restrictions apply for all (for earliest entry) and may depend on institutional subscription	No time restrictions (full *post hoc* population of records)
Access	Via an online platform for which a subscription is often required (e.g. Proquest)	Service provided purely through a free-to-access online search engine
Inclusion	Typically selectively included according to a predefined list of journals, publishers or subject areas	Anything that matches a set of criteria automatically included. Criteria (for Google Scholar): 1) must have a dedicated page with a title, 2) title must be closely followed by authorship list, 3) manuscript should be PDF, HTML or DOC file, 4) manuscript file should include a ‘References’ or ‘Bibliography’ section
Update frequency	Variable–may be as often as weekly, but some databases are monthly or less frequent (e.g. Biological Abstracts, 6 weeks). Updates are based on print versions of journals so will not include ‘early view’ manuscripts until they appear in print. Updates are based on citations submitted by catalogued journals	Typically 1–2 weeks
Examples	Web of Science, Biological Abstracts	Google Scholar, Microsoft Academic Search
Search facility	Full Boolean strings allowed	Variable–Google Scholar allows limited Boolean operators (no nesting using parentheses permitted) and search string limited to 256 characters
Results displayed	Unlimited results from within the database returned, but numbers estimated for large record sets (> c. 5,000). Results sortable by many different fields	Typically limited–Google Scholar limited to first 1,000 with no explanation of or alteration to sort order

According to Thomson Reuters, the Web of Science Core Collections citation database contains almost 50 million research records (http://wokinfo.com/citationconnection/realfacts/; February 2015), with Microsoft Academic Search reporting to catalogue in excess of 45 million records as of January 2013 (http://academic.research.microsoft.com/About/help.htm#9). Google Scholar does not report the volume of citations identifiable via their search facility, although attempts have been made to estimate this that suggest between 1.8 million [3] and 100 million records [4] are identifiable.

“Grey literature” is the term given to describe documents not published by commercial publishers, and it may form a vital component of evidence reviews such as systematic reviews and systematic maps [5], rapid evidence assessments [6] and synopses [7]. Grey literature includes academic theses, organisation reports, government papers, etc. and may prove highly influential in syntheses, despite not being formally published in the same way as traditional academic literature e.g. [8]. Considerable efforts are typically required within systematic reviews to search for grey literature in an attempt to include practitioner-held data and also account for possible *publication bias* [5,9]. Publication bias is the tendency for significant, positive research to be more likely to be published than non-significant or negative research, leading to an increased likelihood of overestimating effect sizes in meta-analyses and other syntheses [10]. The inclusion of grey literature is a central tenet of systematic review methodology, which aims to include all available documented evidence and reduce susceptibility to bias.

Academic citation databases are often the first port of call for researchers looking for information. However, access to databases is often expensive; some costing c. £100,000 per annum for organisations of up to 100 employees. Increasingly, researchers are using academic citation search engines to find information (Haddaway, unpublished data). Academic citation search engines appear to represent an attractive alternative to costly citation databases, cataloguing research almost immediately and not restricting results to certain journals, publishers or subject categories. Search engines are particularly attractive to systematic reviewers, since they have the potential to be used to search for grey literature quickly and simply using one search facility rather than a plethora of individual websites [5].

There is on-going debate regarding the utility of Google Scholar as an academic resource e.g. [11,12], but also as a replacement for traditional academic citation databases and in searches for grey literature in systematic reviews [13,14]. Google Scholar represents an attractive resource for researchers, since it is free-to-use, appears to catalogue vast numbers of academic articles, allows citations to be exported individually, and also provides citation tracking (although see criticism of citation tracking by Delgado Lopez-Cozar et al. [15]). Google Scholar is also potentially useful in systematic reviews, since reliance on just one such platform for searches would: i) offer resource efficiency, ii) offer cost efficiency, iii) allow rapid linking to full texts, iv) provide access to a substantial body of grey literature as well as academic literature, and v) be compatible with new methods for downloading citations in bulk that would allow for a very transparent approach to searching [16].

Previous research has shown that articles identified within systematic reviews are identifiable using Google Scholar [13]. However, other authors have suggested that this does not make Google Scholar an appropriate replacement for academic citation databases, as, in practice, there are considerable limitations in the search facility relative to those of academic databases [11], and there is on-going debate about Google Scholar’s place in research [12]. Shultz [17] listed many limitations that have been attributed to Google Scholar, including that the service permits use of only basic Boolean operators in search strings, which are limited to 256 characters, and that users cannot sort results (although some of the other cited disadvantages have been corrected in recent updates). Two further limitations to the use of Google Scholar in academic searches are the inability to directly export results in bulk as citations (although a limited number of individual citations can be extracted within a set time period) and the display of only the first 1,000 search records with no details of the means by which they are ordered.

Web-based academic search engines, such as Google Scholar, are often used within secondary syntheses (i.e. literature reviews, meta-analyses and systematic reviews). Systematic reviews typically screen the first 50 to 100 search records within Google Scholar e.g. [18,19,20], sometimes restricting searches to title rather than full-text searches e.g. [21]. Such activities are not themselves evidence-based, however. Little is known about how these results are ordered, or what proportion of search results are traditional academic relative to grey literature. Furthermore, this small degree of screening (50 to 100 records) is a very small proportion of the volume of literature found through other sources (often 10s of thousands of records).

Google Scholar has improved greatly in recent iterations; evident from early critiques of the service relative to academic citation databases that cite problems that no longer exist e.g. [22,23]. Whilst the debate on the usefulness of Google Scholar in academic activities has continued in recent years, some improvements to the service offer unequivocal utility; for example, Shariff et al. [24] found that Google Scholar provided access to almost three times as many articles free of charge than PubMed (14 and 5%, respectively).

Any recommendations in systematic review guidance that are made regarding the allocation of greater resources to the use of academic search engines, such as Google Scholar, should be based on knowledge that such resources are worthwhile, and that academic search engines provide meaningful sources of evidence, and do not correspond to wasted effort.

Here, we describe a study investigating the use of Google Scholar as a source of research literature to help answer the following questions:

1. What proportion of Google Scholar search results is academic literature and what proportion grey literature, and how does this vary between different topics?
2. How much overlap is there between the results obtained from Google Scholar and those obtained from Web of Science?
3. What proportion of Google Scholar and Web of Science search results are duplicates and *what causes this duplication*?
4. Are articles included in previous environmental systematic reviews identifiable by using Google Scholar alone?
5. Is Google Scholar an effective means of finding grey literature relative to that identified from hand searches of organisational websites?

## Methods

Seven published systematic reviews were used as case studies [20,25,26,27,28,29,30] (see Table 2). These reviews were chosen as they covered a diverse range of topics in environmental management and conservation, and included interdisciplinary elements relevant to public health, social sciences and molecular biology. The importance and types of grey literature vary between subjects, and a diversity of topics is necessary for any assessment of the utility of a grey literature search tool. The search strings used herein were either taken directly from the string used in Google Scholar in each systematic review’s methods or were based on the review’s academic search string where Google Scholar was not originally searched. Search results in Google Scholar were performed both at “full text” (i.e. the entire full text of each document was searched for the specified terms) and “title” (i.e. only the title of each document was searched for the specified terms) level using the advanced search facility (see https://scholar.google.se/intl/en/scholar/help.html#searching for further details). Searches included patents and citations. Since Google Scholar displays a maximum of 1,000 search results this was the maximum number of citations that could be extracted using the specially developed method described below.

**Table 2:** Systematic reviews (SRs) used as case studies and their search strings (along with modifications to WoS search strings necessary to function in Google Scholar advanced search facility as indicated by strikethrough text). Searches were performed on 06/02/15. Web of Science includes the following databases as part of the MISTRA EviEM subscription; KCI-Korean Journal Database, SciELO Citation Index and Web of Sciences Core Collection.

Systematic review title and reference	Search string	Original systematic review methods	Google scholar search results	Web of Science search results
Evaluating effects of land management on greenhouse gas fluxes and carbon balances in boreo-temperate lowland peatland systems (SR1) [25]	peat AND (“greenhouse gas” OR GHG OR CO2 OR "carbon dioxide" OR CH4 OR N2O OR methane OR "nitrous oxide" OR DOC OR carbon)	Full search in WoS, SR searched first 50 records in Google Scholar	318,000 (full text), 1,120 (title)	4,151 (topic), 419 (title)
Systematic review of effects on biodiversity from oil palm production (SR2) [26]	“oil palm” AND tropic* AND (diversity OR richness OR abundance OR similarity OR composition OR community OR deforestation OR “land use change” OR fragmentation OR “habitat loss” OR connectivity OR “functional diversity” OR ecosystem OR displacement)	Full search in WoS, SR searched first 50 records in Google Scholar	126,000 (full text), 968 (title)	290 (topic), 3 (title)
Which components or attributes of biodiversity influence which dimensions of poverty? (SR3) [20]	(biodiversity OR wildlife) AND (poverty OR livelihoods OR poor)	Full search in WoS, SR searched first 50 records in Google Scholar (term ‘wildlife’ removed from our search as multiple OR sub-strings not possible in Google Scholar)	835,000 (full text), 591 (title)	4,435 (topic), 114 (title)
Evaluating the biological effectiveness of fully and partially protected marine areas (SR4) [27]	marine AND (reserve OR "protected area" OR sanctuary OR "harvest refuge")	Full search in WoS, SR searched first 50 records in Google Scholar	554,000 (full text), 4,310 (title)	47,932 (topic), 1,303 (title)
Human well-being impacts of terrestrial protected areas (SR5) [28]	"protected area" AND (poverty OR “human OR well*” OR socioeconomic* OR econom* OR “human OR health” OR livelihood OR “social OR capital” OR “social OR welfare” OR empowerment OR equity OR “ecosystem OR service” OR perception OR attitude)	Full search in WoS, GS search not performed	49,700 (full text), 68 (title)	1,059 (topic), 122 (title)
Evidence on the environmental impacts of farm land abandonment in high altitude/mountain regions: a systematic map (SR6) [29]	abandonment AND (grassland OR farm OR cropland OR agriculture OR land OR pasture)	Full search in WoS, SR searched first 260 records in Google Scholar	216,000 (full text), 517 (title)	2,550 (topic), 180 (title)
A systematic review of phenotypic responses to between-population outbreeding (SR7) [30]	depression AND ("out-breeding" OR outcrossing OR "out-crossing" OR "out-mating" OR outmating)	Full search in WoS, GS search not performed	15,200 (full text), 50 (title)	1,071 (topic),31 (title)

### 1. What proportion of Google Scholar search results is grey literature?

A download manager (DownThemAll!; http://www.downthemall.net) and web-scraping programme (Import.io; http://www.import.io) were used to download each page of search results (to a maximum of 100 pages; 1000 results) and then extract citations as patterned data from the locally stored HTML files into a database. Two databases (one for the title only search and one for the full text search) for each of the 7 systematic reviews were created, each holding up to 1,000 Google Scholar citations (see S1 File).

Exported citations were assessed and categorised by NRH and AMC as one of the following types of literature:

- ‘Black’–peer-reviewed articles published in academic journals
- ‘Book’–monographs or complete books produced by commercial publishers
- ‘Book chapter’–chapters within books produced by commercial publishers
- ‘Patent’–registered patents and patent applications with the United States Patent and Trademark Office (USPTO)
- ‘Thesis’–dissertations from postgraduate degrees (master’s and doctorates)
- ‘Conference’–presentations, abstracts, posters and proceedings from conferences, workshops, meetings, congresses, symposia and colloquia
- ‘Other’–all other literature that may or may not be peer-reviewed, including; reports, working papers, self-published books, etc.
- ‘Unclear’–any search record that could not be categorised according to the above classification (ambiguous citations were discussed by the reviewers and classed as ‘unclear’ if no consensus could be reached due to limited information).

Book chapters are a subcategory of books but have been separated for additional clarity. These categories have been chosen because they reflect the type of information returned by Web of Science (‘black’ literature) and Google Scholar (all literature). The categories also reflect the emergent classifications that were possible based on information in the citations and any associated descriptions.

For each search type (title or full text) the proportion of literature types across the search results was summarised per page of results to assess the relative location of the types within the results.

### 2. How much overlap is there between Google Scholar and Web of Science?

For each of the 7 systematic review case studies title and full text searches were performed in Google Scholar and Web of Science (25/01/2015) and citation records extracted (all records for Web of Science or the first 1,000 for Google Scholar). Full text search results were not extracted for SR4 since over 47,000 records were returned, which was deemed too expansive for this assessment. The search results were then compared using the fuzzy duplicate identification add-in for Excel described below to investigate the degree of overlap between Web of Science and the first 1,000 Google Scholar search results.

### 3. What proportion of Google Scholar and Web of Science search results are duplicates and what causes this duplication?

Duplicate records are multiple citations that refer to the same article. They are disadvantageous in search results since they do not represent truly unique records and require time and resources for processing. Duplicates also lead to a false estimation of the size of search results: depending on the level of duplication there may be a significant deviation from the true size of search results. The fourteen databases from the 7 case study systematic reviews described above were screened for Google Scholar duplicates using the Excel Fuzzy Duplicate Finder add-in (https://www.ablebits.com/excel-find-similar/) set to find up to 10 character differences between record titles. Potential duplicates were then manually assessed and reasons for duplication (e.g. spelling mistakes or grammatical differences) were recorded.

Searches were performed using Web of Science (using Bangor University’s subscription consisting of Biological Abstracts, MEDLINE, SciELO, Web of Science Core Collections and Zoological Record) using the same 7 search strings used with the above case studies in Google Scholar for topic words. The first 1,000 search results were extracted and assessed for duplicates on title using the Fuzzy Duplicate Finder as described above. Search results were extracted for records ordered both by relevance and by publication date (newest first), with the exception of SR2, SR5 and SR7, where totals of 230, 1,058 and 1,071 records respectively (all returned) were obtained and extracted in full.

### 4. Are articles included in previous environmental systematic reviews identifiable using Google Scholar?

In order to examine the coverage of Google Scholar in relation to studies included in environmental management systematic reviews, the lists of included articles following full text assessment were extracted from six reviews (four SRs described in Table 2; SR1, SR4, SR5, SR6 and two additional reviews; [8,31]) and each record’s title was searched for using Google Scholar. The option in Google Scholar to include citations was selected. Where titles were not found immediately, quotation marks were used, followed by partial removal of the title where possible typographical errors or punctuation variations might cause a record not to be found. Where records were identified as citations (i.e. Google Scholar found a reference within the reference list of another article) this was also recorded. In addition, references from the final lists of included article for three systematic reviews (SR1, SR4, SR6) were searched for in Web of Science as described for Google Scholar, above.

### 5. Is Google Scholar an effective means of finding grey literature identified from hand searches of organisational websites?

For another systematic review search string (SR5, Table 2) the 84 articles that were identified during searches for grey literature in the published review [28] from 16 organisational web sites (see S1 Table) were used to test the ability of Google Scholar to find relevant grey literature using a single search string. The 84 articles were checked against the exported search results for both title and full text searches in Google Scholar (see Methods Section 1 above). The 84 articles were then screened in Google Scholar individually to assess whether they were included in the search engine’s coverage.

## Results

### 1. What proportion of Google Scholar search results is grey literature

**Fig 1:**
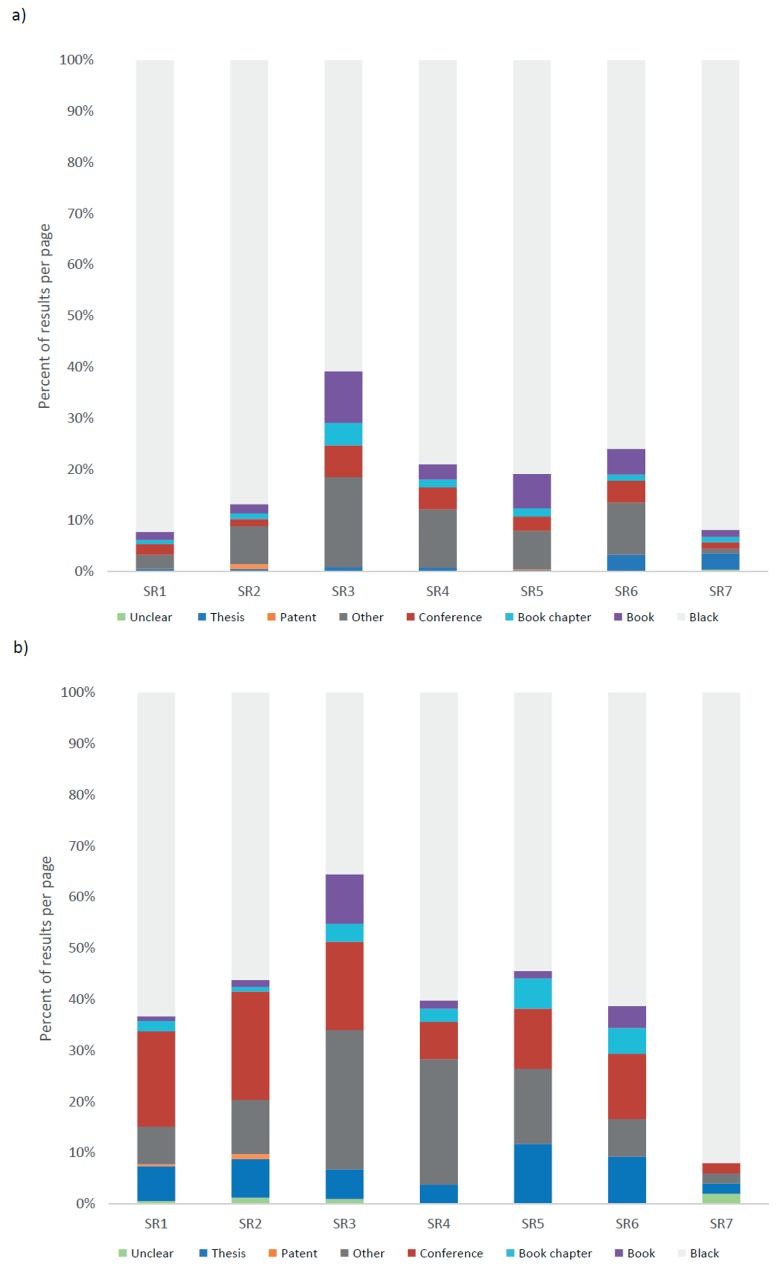
Proportion of total a) full text and b) title Google Scholar search results by literature type for 7 case studies (see Table 2 for descriptions of SR codes).

Between 8 and 39% of full text search results from Google Scholar were classed as grey literature (mean ± SD: 19% ± 11), and between 8 and 64% of title search results (40% ± 17). Fig 1 displays search results by grey literature category, showing a greater percentage of grey literature than academic literature in title search results (43.0%) than full text results (18.9%). Conference proceedings, theses and “other” grey literature (i.e. reports and white-papers) accounted for the increase in the proportion of grey literature in title searches relative to full text searches. Theses formed a particularly small proportion of the full text search results across all case studies (1.3%), but formed a larger proportion of title search results (6.4%). Similarly, conference proceedings were less common in full text search results (3.2%) than title search results (15.3%). The proportion of patents, book chapters and books was similar in full text and title searches (0.2 and 0.3; 1.7 and 2.5; 4.2 and 2.8% respectively).

When examining the location of literature categories across search results (see S1 Fig) several patterns emerge. “Peak” grey literature content (i.e. the point at which the volume of grey literature per page of search results was at its highest and where the bulk of grey literature is found) occurred on average at page 80 (±15 (SD)) for full text results, whilst it occurred at page 35 (± 25 (SD)) for title results. Before these points in the search results grey literature content was low in relative terms. For the majority of the case studies it was not until page 20 to 30 that grey literature formed a majority of each page of search results.

### 2. How much overlap is there between Google Scholar and Web of Science?

Google Scholar demonstrated modest overlap with Web of Science title searches: this overlap ranged from 10 to 67% of the total results in Web of Science (Table 3). The overlap was highly variable between subjects, with reviews on marine protected area efficacy and terrestrial protected area socioeconomic impacts demonstrating the lowest overlap (17.1 and 10.3% respectively). Two case study title searches returned more than the viewable limit of 1,000 search results in Google Scholar (SR1 and SR4) and so only the first 1,000 could be extracted.

**Table 3:** Overlap between Web of Science (WoS) and Google Scholar (GS) for title searches in Web of Science and the first 1,000 search results from title searches in Google Scholar. See Table 2 for case study explanations.

Case Study	Number of overlapping search results (% of WoS records)	No. of WoS title search results	No. of GS title search results
SR1	157 (37.8%)	415	1,120
SR2	2 (66.7%)	3	968
SR3	32 (49.2%)	65	591
SR4	223 (17.1%)	1,301	4,310
SR5	6 (10.3%)	58	68
SR6	68 (37.8%)	180	517
SR7	18 (58.1%)	31	50

Full text search results from Google Scholar demonstrated low overlap with Web of Science results (Table 4), ranging from 0.2 to 19.8% of the total Web of Science results.

**Table 4:** Overlap between Web of Science (WoS) and Google Scholar (GS) for topic word searches in Web of Science and the first 1,000 search results from full text searches in Google Scholar. n/a corresponds to search results that were too voluminous to download in full. See Table 2 for case study explanations.

Case Study	No. of WoS topic word search records	No. of GS full text search records	Number of overlapping search results (% of WoS records)
SR1	4,504	318,000	255 (5.7%)
SR2	230	126,000	11 (4.8%)
SR3	4,240	835,000	106 (2.5%)
SR4	47,932	554,000	n/a
SR5	1,059	49,700	87 (8.2%)
SR6	2,549	216,000	5 (0.2%)
SR7	1,071	15,200	212 (19.8%)

### 3. What proportion of Google Scholar and Web of Science search results are duplicates and how do these duplicates come about?

Duplication rates (i.e. the percentage of total results that are duplicate records) for Google Scholar and Web of Science are shown in Table 5 and range from 0.00 to 2.93%. Rates of duplication are substantially higher within Google Scholar than Web of Science, and rates are far higher in title searches within Google Scholar than full text searches (Table 6), although this is quite variable between the 7 case studies (1.0 to 4.8%%).

**Table 5:** Duplication rates (proportion of total results that are duplicates) for Google Scholar and Web of Science for title-level, topic word and full text searches using 7 case study systematic review search strings. Numbers in parentheses correspond to the standard deviations of the individual case study duplication rates. Sample size refers to the number of search records in total, followed by the number of independent search strings (i.e. the number of case studies investigated).

Search Platform	Search Type	Duplication Rate (mean %)	Sample Size (search records, search strings)
Google Scholar	Full text	0.56 (± 0.59)	6988, 7
	Title	2.93 (± 1.47)	4194, 7
Web of Science	Topic words (sorted by publication date, newest first)	0.00 (± 0.00)	6359, 7
	Topic words (sorted by relevance)	0.03 (± 0.05)	4000, 4
	Title	0.05 (± 0.03)	2102, 7

**Table 6:** Duplication rates (proportion of total results that are duplicates) in Google Scholar and Web of Science searches across the 7 case studies. Duplication rates are assessed for up to 1,000 search records (or the total number where less than c. 1,300). For Web of Science the full text results were ordered by publication date (newest first) and relevance where more than 1,000 results were returned. Numbers are duplication rate (%) followed by total search records in parentheses.

	Google Scholar		Web of Science		
Case Study	Title Search	Full Text Search	Title Search	Full Text Search (publ. date)	Full Text Search (relevance)
Evaluating effects of land management on greenhouse gas fluxes and carbon balances in boreo-temperate lowland peatland systems (SR1) [25]	3.4 (1000)	0.2 (998)	0 (415)	0 (1000)	0 (1000)
Systematic review of effects on biodiversity from oil palm production (SR2) [26]	2.6 (968)	0.2 (990)	0 (3)	0 (230)	n/a
Which components or attributes of biodiversity influence which dimensions of poverty? (SR3) [20]	4.4 (591)	0.7 (1000)	0 (114)	0 (1000)	0 (1000)
Evaluating the biological effectiveness of fully and partially protected marine areas (SR4) [27]	1.0 (1000)	0.0 (1000)	0.1 (1301)	0 (1000)	0.1 (1000)
Human well-being impacts of terrestrial protected areas (SR5) [28]	1.5 (68)	0.1 (1000)	0 (58)	0 (1058)	n/a
Evidence on the environmental impacts of farm land abandonment in high altitude/mountain regions: a systematic map (SR6) [29]	4.8 (517)	1.3 (1000)	0 (180)	0 (1000)	0 (1000)
A systematic review of phenotypic responses to between-population outbreeding (SR7) [30]	4.0 (50)	1.4 (1000)	0 (31)	0 (1071)	n/a

Duplicates appear to have arisen for a range of reasons. First, typographical errors introduced by manual transcription were found in both Google Scholar (15% of title records) and Web of Science. For example, the sole example of a duplicate from Web of Science is that of the two records that differ only in the spelling of the word ‘Goukamma’ (or Goukarmma) in the following title: “A change of the seaward boundary of Goukamma Marine Protected Area could increase conservation and fishery benefits”. Differences in formatting and punctuation are a subset of typographical errors and corresponded to 18% of title level duplicates. Second, capitalisation causes duplication in Google Scholar, and was responsible for 36% of title level duplicates. Third, incomplete titles (i.e. some missing words) were responsible for 15% of title level duplicates. Fourth, automated text detection (i.e. when scanning documents digitally) was responsible for 3% of title level duplicates. Fifth, Google Scholar also scans for citations within references of selected included literature, and the presence of both these citations and the original articles themselves was responsible for 13% of title level duplication.

### 4. Are articles included in previous environmental systematic reviews identifiable using Google Scholar?

Many of the included articles from the six published systematic review case studies were identified when searching for those articles specifically in Google Scholar (Table 7). However, a significant proportion of studies in one review [31] were not found at all using Google Scholar (31.5%). Other reviews were better represented by Google Scholar coverage (94.3 to 100% of studies). Only one review had an included article list that was fully covered by Google Scholar, the review with the smallest evidence base of only 37 studies [31]. For those reviews where studies were not identified by Google Scholar, a further search was performed for these missing studies in Web of Science (Table 7), which demonstrated that some of these studies (6 studies from 2 case study reviews) were catalogued by Web of Science.

**Table 7:** The ability of Google Scholar to find included articles from six published systematic reviews. Records identified as citations are found only within reference lists of other articles (their existence is not verified by the presence of a publisher version or full text article, unlike hyperlinked citations).

Review	Identified	Identified (as citation)	Not identified	(Of which, findable in WoS^1^)
Evaluating effects of land management on greenhouse gas fluxes and carbon balances in boreo-temperate lowland peatland systems (SR1) [25]	59	0	1	0
Evaluating the biological effectiveness of fully and partially protected marine areas (SR4) [27]	158	24	11	3
Human well-being impacts of terrestrial protected areas (SR5) [28]	162	4	10	3
Evidence on the environmental impacts of farm land abandonment in high altitude/mountain regions: a systematic map (SR6) [29]	180	4	1	0
What are the impacts of reindeer/caribou (Rangifer tarandus L.) on arctic and alpine vegetation? [31]	35	2	0	0
What is the influence on water quality in temperate eutrophic lakes of a reduction of planktivorous and benthivorous fish? [8]	77	8	39	0

^1^ For those articles not found using Google Scholar, Web of Science searches were carried out using Bangor University subscription (Biological Abstracts, MEDLINE, SciELO Citation Index, Web of Science Core Collections, Zoological Record).

Google Scholar search results that were available only as citations (i.e. obtained from the reference lists of other search results) constituted between 0 and 15.2% of identified results. Citations typically do not lead to web pages that provide additional information and cannot therefore be verified manually by users.

When searching specifically for individual articles, Google Scholar catalogued a larger proportion of articles than Web of Science (% of total in Google Scholar / % of total in Web of Science: SR1, 98.3/96.7; SR4, 94.3/83.9; SR6, 99.4/89.7).

### 5. Is Google Scholar an effective means of finding grey literature identified from hand searches of organisational websites?

None of the 84 grey literature articles identified by SR5 [28] were found within the exported Google Scholar search results (68 total records from title searches and 1,000 of a total 49,700 records from full text searches). However, when searched for specifically 61 of the 84 articles were identified by Google Scholar.

## Discussion

This paper set out to investigate the role of Google Scholar in searches for academic and grey literature in systematic and other literature reviews. There is much interest in Google Scholar due to its free-to-use interface, apparent comprehensiveness e.g. [11,12,13,14], and application within systematic reviews [16]. However, previous studies have disagreed on whether the service could be used as a standalone resource e.g. [11,12]. Our study enables recommendations to be made for the use of Google Scholar in systematic searches for academic and grey literature, particularly in systematic reviews.

### 1. What proportion of Google Scholar search results is grey literature?

Our results show that Google Scholar is indeed a useful platform for searching for environmental science grey literature that would benefit researchers such as systematic reviewers, agreeing with previous research in medicine [32,33]. Our investigations also demonstrate that more grey literature is returned in title searches than full text searches (43% relative to 19%, respectively), slightly more than previously found in an investigation of full text searching alone in an early version of Google Scholar (13% of total results; [17]). The grey literature returned by Google Scholar may be seen by some as disadvantageous given its perceived lack of verification (through formal academic peer-review), particularly where researchers are looking for purely traditional academic evidence. However, this may be particularly useful for those seeking evidence from across academic and grey literature domains; for example, those wishing to minimise the risk of publication bias (the over-representation of significant research in academic publications [34]).

We found that the greatest volume of grey literature in searches occurs at around page 35 for title searches. This finding indicates that researchers, including systematic reviewers, using Google Scholar as a source of grey literature should revise the current common practice of searching the first 50–100 results (5–10 pages) in favour of a more extensive search that looks further into the records returned. Conversely, those wishing to use title searching for purely academic literature should focus on the first 300 results to reduce the proportion of grey literature in their search results.

The grey literature returned in the 7 systematic review case studies examined herein mostly consisted of “other” grey literature and conference proceedings; i.e. white papers and organisational reports. Reports and white papers may prove particularly useful for secondary syntheses, since they may often represent resources that are commissioned by policy and practice decision-makers. Conference proceedings typically represent academic works that have not been formally published in commercial academic journals: such articles may also provide useful evidence for reviewers, particularly systematic reviewers. Academic theses were more common in title searches in Google Scholar, whilst books were more common in full text searches. Theses can provide a vital source of grey literature [35], research that never makes it into the public domain through academic publications. It is worth noting that whilst academic peer-review is not a guarantee of rigour, research that has not been through formal academic peer-review should be carefully appraised before being integrated into syntheses such as systematic reviews [5]. Google Scholar may thus prove to be a useful resource in addition to dedicated databases of theses (e.g. DART-Europe; http://www.dart-europe.eu/basic-search.php) and other grey literature repositories (e.g. ProceedingsFirst; https://www.oclc.org/support/services/firstsearch/documentation/dbdetails/details/Proceeding.en.html).

### 2. How much overlap is there between Google Scholar and Web of Science?

Surprisingly, we found relatively little overlap between Google Scholar and Web of Science (10–67% of WoS results were returned using searches in Google Scholar using title searches). For the largest set of results (SR4) only 17% of WoS records were returned in the viewable results in Google Scholar (restricted to the first 1,000 records). However, the actual number of returned results in Google Scholar was 4,310, with only the first 1,000 being viewable due to the limitations of Google Scholar. Assuming an even distribution of overlapping studies across these results we might expect a modest 73% coverage in total (calculated by applying a consistent rate of 17% from the first 1,000 to the full set of 4,310 search records). The limitations of viewable results in Google Scholar make an assessment of overlap impossible when the number of results is greater than 1,000. The case study SR1 only slightly exceeded the viewable limit of 1,000 studies and identified an overlap of 38%, however.

The relatively low overlap between the two services demonstrates that Google Scholar is not a suitable replacement for traditional academic searches: although its results are greater than those in Web of Science, the majority of Web of Science search results are not returned by Google Scholar. However, Google Scholar is a useful addition to traditional database searching, since a large body of search records was returned for each case study that did not overlap, potentially increasing the coverage of any multi-database search, such as those carried out in systematic reviews.

### 3. What proportion of Google Scholar and Web of Science search results are duplicates and how do these duplicates come about?

Duplicates within citation databases are disadvantageous because they represent false records. Although the individual reference may be correct, its presence in the database contributes to the number of results. Where large numbers of references must be screened manually, as in systematic reviews, duplicates may also represent a waste of resources where they are not automatically detectable. Duplication rates in Web of Science were very low (0–0.05%), but notably higher in Google Scholar (1–5%). Duplication in Google Scholar occurred as a result of differences in formatting, punctuation, capitalisation, incomplete records, and mistakes during automated scanning and population of the search records. The sensitivity of Google Scholar searches comes at a cost, since identical records are identified as unique references. This may not be a significant problem for small-scale searches, but a 5% duplication rate represents a substantial waste of resources in a systematic review where tens of thousands of titles must be screened manually.

### 4. Are articles included in previous environmental systematic reviews identifiable using Google Scholar?

Gehano et al. [13] found that Google Scholar was able to identify all 738 articles from across 29 systematic reviews in medicine, and concluded that it could be used as a standalone resource in systematic reviews, stating that “if the authors of the 29 systematic reviews had used only GS, no reference would have been missed”. As pointed out by other researchers e.g. [14], this conclusion is incorrect, since the ability to find specific, known references does not equate to an ability to return these references using a search strategy as might be conducted within a systematic review: most importantly, the relevant articles may be returned outside of the viewable 1,000 records. Giustini and Boulos [14] found that 5% of studies from a systematic review could not be identified using specific searches in Google Scholar, whilst Boeker et al. [11] found that up to 34% of studies from 14 systematic reviews were missed.

Google Scholar was able to find much of the existing literature included within the systematic review case studies in our investigations, and indeed found more than Web of Science in the three case studies examined. As such, Google Scholar provides a powerful tool for identifying articles that are already known to exist (for example, when looking for a citation or access to a full text document). In addition, the search engine was also able to identify large amounts of potentially relevant grey literature. However, some important evidence was not identified at all by Google Scholar (31.5% in one case study), meaning that the review may have come to a very different conclusion if it had relied solely on Google Scholar. Similarly, Web of Science alone is insufficient to identify all relevant literature. As described above, Google Scholar may provide a useful source of evidence in addition to traditional academic databases, but it should not be used as a standalone resource in evidence-gathering exercises such as systematic reviews.

### 5. Is Google Scholar an effective means of finding grey literature identified from hand searches of organisational websites?

Google Scholar was able to identify a large proportion of the grey literature found in one case study through hand searching of organisational websites (61 of 84 articles). However, 23 articles could not be found using the search engine. Furthermore, the 61 articles found were not returned when using a typical systematic review-style search string. Together, these factors demonstrate that Google Scholar is a useful resource in addition to hand searching of organisational websites, returning a large volume of potentially relevant information, but that it should not be used as a standalone resource for grey literature searching, since some vital information is missed. Hand searching, as recommended by the Collaboration for Environmental Evidence Guidelines in Systematic Reviews [5], is restricted only to those websites included in an *a priori* protocol. Google Scholar exhaustively searches the internet for studies, however, and whilst it may be more coarse than fine-level hand searching (i.e. missing studies), the addition of a Google Scholar search targeting grey literature would increase comprehensiveness without giving cause for concern with relation to any systematic bias. However, since the algorithms that order search results are not disclosed, a substantial proportion of search results should be examined.

## Other Considerations

As mentioned above, only the first 1,000 search results can be viewed in Google Scholar, and the order in which results are returned is not disclosed. Furthermore, the ‘advanced’ search facility supports only very basic Boolean logic, accepting only one set of ‘OR’ or ‘AND’ arguments, not both. In addition, variations in the way that subscript and superscript text, for example with chemical symbols, are displayed and recognised mean that poor matching occurs during searches where these characters form part of article titles. Finally, Google Scholar has a low threshold for repetitive activity that triggers an automated block to a user’s IP address (in our experience the export of approximately 180 citations or 180 individual searches). Thankfully this can be readily circumvented with the use of IP-mirroring software such as Hola (https://hola.org/), although care should be taken when systematically accessing Google Scholar to ensure the terms of use are not violated.

## Conclusions

We have provided evidence that Google Scholar is a powerful tool for finding specific literature, but that it cannot be a replacement for traditional academic citation databases, nor can it replace hand-searching for grey literature. The limitations of the number of search results displayed, the incomplete Boolean operation of the advanced search facility, and the non-disclosure of the algorithm by which search results are ordered mean that Google Scholar is not a transparent search facility. Moreover, the high proportion of grey literature that is missed by Google Scholar mean that it is not a viable alternative to hand searching for grey literature as a stand-alone tool. Despite this, Google Scholar is able to identify a large body of additional grey literature in excess of that found by either traditional academic citation databases or grey literature identification methods. These factors make Google Scholar an attractive supplement to hand searching, further increasing comprehensiveness of searches for evidence.

We also note that the development of tools to take snapshots of search results from Google Scholar and extract these results as citations can significantly increase the efficiency and transparency of using Google Scholar (i.e. beyond the arbitrary first 50 search results currently favoured in many systematic reviews).

Several recommendations can be made based on our findings for those wishing to use Google Scholar as a resource for research evidence:

1. Finding: Google Scholar is capable of identifying the majority of evidence in the systematic review case studies examined when searching specifically for known articles.
Recommendation: Google Scholar is a powerful, free-to-use tool that can be recommended if looking for specific research studies.
2. Finding: Google Scholar is not capable of identifying all relevant evidence identified in the systematic review case studies examined, missing some vital information (as did Web of Science).
Recommendation: Google Scholar (and Web of Science) should not be used as standalone resources for finding evidence as part of comprehensive searching activities, such as systematic reviews.
3. Finding: Substantially more grey literature is found using title searches in Google Scholar than full text searches.
Recommendation: If looking for grey literature, reviewers should consider using title searches. If looking for academic literature title searches will yield a great deal of unsuitable information.
4. Finding: Title level searches yield more conference proceedings, theses and ‘other’ grey literature.
Recommendation: Title level searches may be particularly useful in identifying as yet unpublished academic research grey literature as well as organisational reports and government papers [9]
5. Finding: The majority of grey literature begins to appear after approximately 20 to 30 pages of results.
Recommendation: If looking for grey literature the results should be screened well beyond the 20^th^ page.

In summary, we find Google Scholar to be a useful supplement in searches for evidence, particularly grey literature so long as its limitations are recognised. We recommend that the arbitrary assessment of the first 50 search results from Google Scholar, frequently undertaken in systematic reviews, should be replaced with the practice of recording snapshots of all viewable search results: i.e. the first 1,000 records. This change in practice could significantly improve both the transparency and coverage of systematic reviews, especially with respect to their grey literature components.

## Supporting Information

S1 Fig Google Scholar search results separated by literature type. Search results by page for 7 case studies (see Table 2 for descriptions), for a) full text and b) title searches. Results displayed are for the total number of extractable records in Google Scholar.
(XLSX)

S1 File Google Scholar Search Results. Database of Google Scholar full text and title searches for 7 case study systematic reviews.
(XLSX)

S1 Table Organisational websites list for SR5. List of organisations yielding potentially relevant evidence for a systematic review on the human wellbeing impacts of terrestrial protected areas.
(DOCX)
