# Supplementary material for: The Role of Google Scholar in Evidence Reviews and Its Applicability to Grey Literature Searching
Source: PLoS One. 2015 Sep 17;10(9):e0138237. doi: 10.1371/journal.pone.0138237 (PMC4574933; doi:10.1371/journal.pone.0138237)
Supplement: S1 Table — List of organisations yielding potentially relevant evidence for a systematic review on the human wellbeing impacts of terrestrial protected areas. (DOCX) [file pone.0138237.s003.docx]

Table S2. List of organisations yielding potentially relevant evidence for a systematic review on the human wellbeing impacts of terrestrial protected areas (SR5, [21]).

| **Organisation** | |
| --- | --- |
| CFS website | Nature Valuation website |
| Conservation International website | ODI website |
| DFID website | RECOFTC website |
| Eldis (Livelihoods Connect) website | Rights and Resources website |
| ETFRN website | Tropenbos website |
| GEF EO | UNEP-WCMC website |
| IIED website | USAID website |
| IUCN website | World Bank website |
